# The effects of gesture and action training on the retention of math equivalence

**DOI:** 10.3389/fpsyg.2024.1386187

**Published:** 2024-07-04

**Authors:** Alyssa J. Kersey, Cristina Carrazza, Miriam A. Novack, Eliza L. Congdon, Elizabeth M. Wakefield, Naureen Hemani-Lopez, Susan Goldin-Meadow

**Affiliations:** ^1^Department of Psychology, University of Chicago, Chicago, IL, United States; ^2^Department of Medical Social Sciences, Northwestern University Feinberg School of Medicine, Chicago, IL, United States; ^3^Department of Psychology, Williams College, Williamstown, MA, United States; ^4^Department of Psychology, Loyola University Chicago, Chicago, IL, United States; ^5^Department of Comparative Human Development, University of Chicago, Chicago, IL, United States

**Keywords:** gesture, action, individual differences, math instruction, retention

## Abstract

**Introduction:**

Hand gestures and actions-with-objects (hereafter ‘actions’) are both forms of movement that can promote learning. However, the two have unique affordances, which means that they have the potential to promote learning in different ways. Here we compare how children learn, and importantly retain, information after performing gestures, actions, or a combination of the two during instruction about mathematical equivalence. We also ask whether individual differences in children’s understanding of mathematical equivalence (as assessed by spontaneous gesture before instruction) impacts the effects of gesture- and action-based instruction.

**Method:**

Across two studies, racially and ethnically diverse third and fourth-grade students (N=142) were given instruction about how to solve mathematical equivalence problems (eg., 2+9+4=__+4) as part of a pretest-training-posttest design. In Study 1, instruction involved teaching students to produce either actions or gestures. In Study 2, instruction involved teaching students to produce either actions followed by gestures or gestures followed by actions. Across both studies, speech and gesture produced during pretest explanations were coded and analyzed to measure individual differences in pretest understanding. Children completed written posttests immediately after instruction, as well as the following day, and four weeks later, to assess learning, generalization and retention.

**Results:**

In Study 1 we find that, regardless of individual differences in pre-test understanding of mathematical equivalence, children learn from both action and gesture, but gesture-based instruction promotes retention better than action-based instruction. In Study 2 we find an influence of individual differences: children who produced relatively few types of problem-solving strategies (as assessed by their pre-test gestures and speech) perform better when they receive action training before gesture training than when they receive gesture training first. In contrast, children who expressed many types of strategies, and thus had a more complex understanding of mathematical equivalence prior to instruction, performed equally with both orders.

**Discussion:**

These results demonstrate that action training, followed by gesture, can be a useful stepping-stone in the initial stages of learning mathematical equivalence, and that gesture training can help learners retain what they learn.

## Introduction

Decades of research and careful observation of human behavior have shown that actions using concrete objects, in which learners are encouraged to feel, move, and interact with the physical world, can be powerful instructional tools (e.g., Montessori, 1909; Piaget, 1953). For instance, when students are asked to perform specific actions with objects during instruction, they learn more than when they do not act. This effect has been demonstrated across a host of science, technology, engineering, and math (STEM) domains (Freeman et al., 2014; Nathan et al., 2014; Smith et al., 2014; Abrahamson and Trninic, 2015; Kontra et al., 2015) and in areas like object recognition (Harman et al., 1999). Gestures––which we define here as hand movements that accompany speech and express meaning but do not have a direct effect on concrete objects––also lead to improved learning outcomes (e.g., Cook et al., 2008; Goldin-Meadow et al., 2009), especially when learners are producing, rather than observing, gesture during a lesson (Dargue et al., 2019).

Gestures and actions-on-objects are similar in that they are both forms of *physical movement* involving the body or the hands. But gestures are distinct from actions-on-objects because they, by definition, do not involve any direct manipulation of physical objects, nor do they create lasting changes in the physical environment. For example, one could gesture about how to unscrew the top of a jar without ever touching a jar. But the action of unscrewing, whether used in a functional or pedagogical context, would involve touching the lid and the jar and actually performing the unscrewing motion to remove the lid. In this sense, gestures *represent* actions, ideas, or objects in an abstract, schematized format (Kita et al., 2017; Novack and Goldin-Meadow, 2017). Actions-on-objects tend not to be representational (although they may be used to teach abstract concepts).

These key differences mean that actions and gestures might be construed differently by learners and thus might have distinct effects on learning. Previous work has, indeed, shown that gesture can lead learners to flexibly generalize their understanding of new material beyond the initial learning context (Novack et al., 2014; Levine et al., 2018; Wakefield et al., 2018a); in contrast, learning through actions-on-objects leads to a more rigid conceptual understanding tied to a particular problem structure or set of objects. At the same time, “hands on” concrete learning experience, as provided through actions-on-objects (e.g., Montessori, 1909), has been shown to help learners who need scaffolding on a task before they take the next step with symbolic or representational tools like gesture or language (Piaget, 1953; Congdon et al., 2018).

A long line of work has shown that asking children to produce gestures along with speech during instruction in mathematics not only promotes acquisition of a novel concept, but also leads to longer lasting learning than asking them to produce speech without gesture (Cook et al., 2008; Goldin-Meadow et al., 2009). Mathematical equivalence, the concept that two sides of an equation must be equal, is a particularly difficult concept for children, especially when the problem follows a non-canonical format; for example, when the “blank” is not the only thing on the right-hand side of the equation (3 + 6 + 5 = __ + 5). The difficulty children have with this concept is a concern because success on missing-addend mathematical equivalence problems has been found to predict later algebra skills, even after controlling for the child’s math achievement (McNeil et al., 2019). Moreover, success in algebra predicts future success in advanced STEM courses (Riley, 1997; Atanda, 1999; Adelman, 2006; Hansen, 2014), and failure is associated with negative outcomes such as dropping out of high school (Allensworth and Easton, 2005; Adelman, 2006).

To date, only one study has directly compared gesture-based instruction to action-based instruction on this key mathematical concept: Gesture-based instruction led to more flexible learning than action-based instruction on an immediate post-test (Novack et al., 2014). More specifically, children in a gesture training condition solved transfer problems correctly (i.e., problems that differed in format from those introduced during instruction); children in an action training condition did not display this flexibility. This study demonstrates the positive effects of gesture on generalization, but leaves many questions unanswered. The study did not test whether gesture-based instruction promotes *retention* (memory over time) of mathematical equivalence learning over and above action-based instruction. Initial problem-solving insight is important, but *retention* of the insight is the goal of learning. Additionally, participants were taught to produce *either* gestures *or* actions on objects, leaving open the possibility that combining the two instructional approaches might be particularly good for learning. Finally, the effects of gesture and action were assessed at the group level and did not examine whether individuals might differ in which method of instruction is best for them.

In Study 1, we directly compare action- and gesture-based instruction on learning mathematical equivalence, but rather than focus on generalization, we focus on retention by offering follow up tests 1 day and 1 month after initial training. Understanding whether one type of instruction helps learning to “stick” more than another has clear practical and theoretical implication.

In Study 2, we turn our focus away from pitting gesture- and action-based instruction against one another and move to a design where all learners are given *both* types of instruction (action and gesture) within a single training session. This design also allows us to test whether the order in which the two instructional approaches are presented affects key metrics of learning.

Finally, in both studies, we assess individual differences in how well mathematical equivalence is understood prior to training, and we examine the impact of those differences on learning.

### Retaining new knowledge after mathematical equivalence instruction

Novack et al. (2014) found that teaching children to produce a correct mathematical equivalence gesture during instruction led to better generalization than teaching them to perform the same strategy using concrete objects (plastic numbers). In Study 1, we ask whether children who are told to gesture retain the knowledge they gained during a math lesson better than children who are told to perform the same movements on plastic numbers.

### Instruction that combines action and gesture

Both Piaget (1953) and Bruner (1966) argued that learning is most effective when children begin with concrete objects and multiple exemplars and then move to a more abstract or symbolic representation. With respect to math learning, Mix (2010) has shown that acting on objects (e.g., using mathematical manipulatives in a classroom) provides a concrete model that serves as a foundation for new skills and concepts. Given that actions-on-objects offer a more concrete instantiation of mathematical equivalence than abstract gestures (Congdon et al., 2017, 2018), this could explain why actions lead to better initial learning (Congdon et al., 2018) *and* why gestures lead to better transfer (Novack et al., 2014).

Fyfe et al. (2015) varied the order in which children received concrete vs. abstract training on mathematical equivalence, and found that children in the most successful training condition started by balancing a physical scale or counting out physical tokens (concrete), then completed a two-dimensional worksheet version of that representation, and finally solved equations using only Arabic numerals (abstract). In our study, if we conceptualize actions-on-objects as more concrete than gestures (Novack et al., 2014; Novack and Goldin-Meadow, 2017), then we should see a greater benefit for instruction that teaches actions *followed by* gesture than instruction that teaches actions *preceded by* gestures. This is the focus of Study 2.

### Examining individual differences

Most research comparing action-based and gesture-based instruction has focused on group level analyses (i.e., comparing one condition to another). But individual differences in learner-profiles might influence how effective a training manipulation is (Congdon et al., 2024). A recent study comparing gesture- and action-based instruction in measurement found that children who used a more advanced strategy prior to training learned equally well from gesture and action training, but children who used a less advanced strategy learned best from action. Action may be a more effective tool than gesture for novice learners (see also Wakefield and James, 2015, who found that producing gesture improved children’s understanding of palindromes, but only for children who already had strong phonological skills).

To explore individual differences in understanding mathematical equivalence prior to training, we turned to an index that has been shown to predict readiness-to-learn mathematical equivalence. Children spontaneously gesture as they explain their answers to pretest equivalence problems. Those whose pretest gestures convey a problem-solving strategy not found in the accompanying speech, that, is, children who produce gesture-speech *mismatches* on a pretest, are more likely to profit from a math lesson than children who do not produce mismatches (Church and Goldin-Meadow, 1986; Perry et al., 1988; Broaders et al., 2007). Moreover, mismatchers produce more different types of strategies at pretest, suggesting that they have more knowledge about the problem than children who do not mismatch (Goldin-Meadow et al., 1993a). As all children solve and explain the pretest problems incorrectly, the additional knowledge that mismatchers display must be implicit––in the sense that they do not articulate it in speech (only in gesture).

Proposals in education (see, for example, Kalyuga, 2007) suggest that instruction should generally be tailored to a learner’s knowledge base. Learners with less knowledge may benefit from methods with concrete instructional support. But learners with more knowledge may be hindered by concrete support, which might prevent them from advancing their knowledge as much as they could have had they been given more abstract instructional support. In exploratory analyses, we ask whether children who are, and are not, implicitly entertaining multiple ways to solve mathematical equivalence problems (as revealed in pretest gesture; Goldin-Meadow et al., 1993a,b) are equally receptive to instruction using gestures or actions (Study 1). We then ask whether children with and without implicit knowledge profit equally from combining the two types of instruction (Study 2).

The goal of Study 1 is to extend previous work comparing the efficacy of gesture-based and action-based instruction by specifically investigating effects on *retention* of mathematical equivalence over a one-month delay. Compared to action-based training, gesture-based training has been shown to lead to larger gains after a 1-week delay on a mental rotation task (Levine et al., 2018), and to better performance on transfer trials after a 24-h delay on a word-learning task (Wakefield et al., 2018a). If improving retention is a domain general effect of gesture-based training, then we would expect it to also lead to better retention of mathematical equivalence than action-based training.

## Study 1: Methods

### Participants

Seventy-one third- and fourth-grade students (M = 9.3 years, SD = 0.6 years; 44 females) who did not correctly solve any problems on a pre-test of mathematical equivalence participated in Study 1. Children were recruited in public elementary schools in a large urban city. The study focused on children of this age because third- and fourth-graders typically do not understand the concept of mathematical equivalence and fail to solve problems of this format (e.g., McNeil, 2014). Participants were racially and ethnically diverse (Race: 28% White, 5.6% Asian, 5.6% Black, 2.8% More than one race, 1.4% Native Pacific Islander, 1.4% Native American, 43.7% unknown or unreported; Ethnicity: 64.8% Hispanic, 22.5% Not Hispanic, 12.7% unknown or unreported). We used the highest level of parent education to index socioeconomic status. Overall, the sample came from lower SES households: 53.8% of parents reported having a high-school degree or less; 20% had completed some college; 26.2% reported having a college or graduate degree (8.5% unknown or unreported). Prior to the study, parents provided written consent and children gave assent. Children received a small prize and certificate of participation, and teachers of participating classrooms received a gift card to a local learning store.

### Materials

During the training phase, math problems were written by the experimenter on a white dry-erase magnetic board, and black magnetic number tiles were placed over each number (see Novack et al., 2014). During the assessments, children were asked to solve three types of missing addend problems (Form A, Form B, and Form C) using pencil and paper at each time point (Pre-test, Post-test, Testing Day 2, and Testing Day 3). Forms A and B were equal addend mathematical equivalence problems––in Form A problems, which were used for training and thus should be thought of as the “easiest” problems for learners to solve, the last addend of the left side was repeated on the right side. In Form B problems, which participants did not see during training but were very similar in structure to Form A, the equal addend was the first number on each side of the equation. In Form C problems, there were no identical addends on the two sides of the equal sign, and the blank could be in either the first or second position on the right side of the equation. Below are examples of each problem type, using similar numbers to facilitate comparison.

Form A: 5 + 6 + 3 = __ + 3.

Form B: 5 + 6 + 3 = 5 + __.

Form C: 5 + 6 + 3 = 2 + __.

### Design and procedure

**Figure 1:**
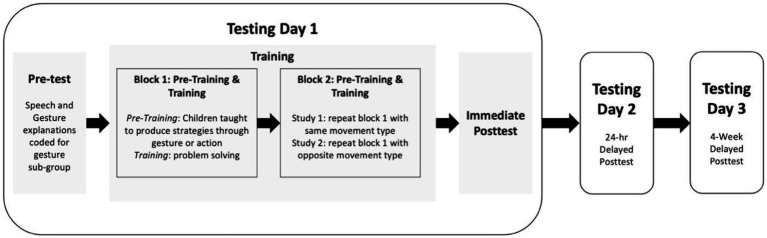
An overview of the procedure used in Study 1 and Study 2.

Children were tested one-on-one by an experimenter on three separate days in a quiet location at their school (Figure 1). Testing Day 1 consisted of a pre-test with verbal explanations, training phase, and immediate post-test with verbal explanations. Children were randomly assigned to one of two training conditions: action or gesture. To measure retention of the instruction material, children also completed paper-and-pencil follow-up tests after a 24-h (Testing Day 2) and four-week (Testing Day 3) delay.

### Testing day 1

**Pre-test**. All children completed a paper-and-pencil pre-test consisting of six missing addend problems (2 Form A; 2 Form B; 2 Form C). The experimenter then wrote each problem with the child’s answer on a white board and asked the child to explain their solutions, one at a time. Children’s explanations were video recorded in order to capture their speech and gesture strategies.^1^

**Figure 2:**
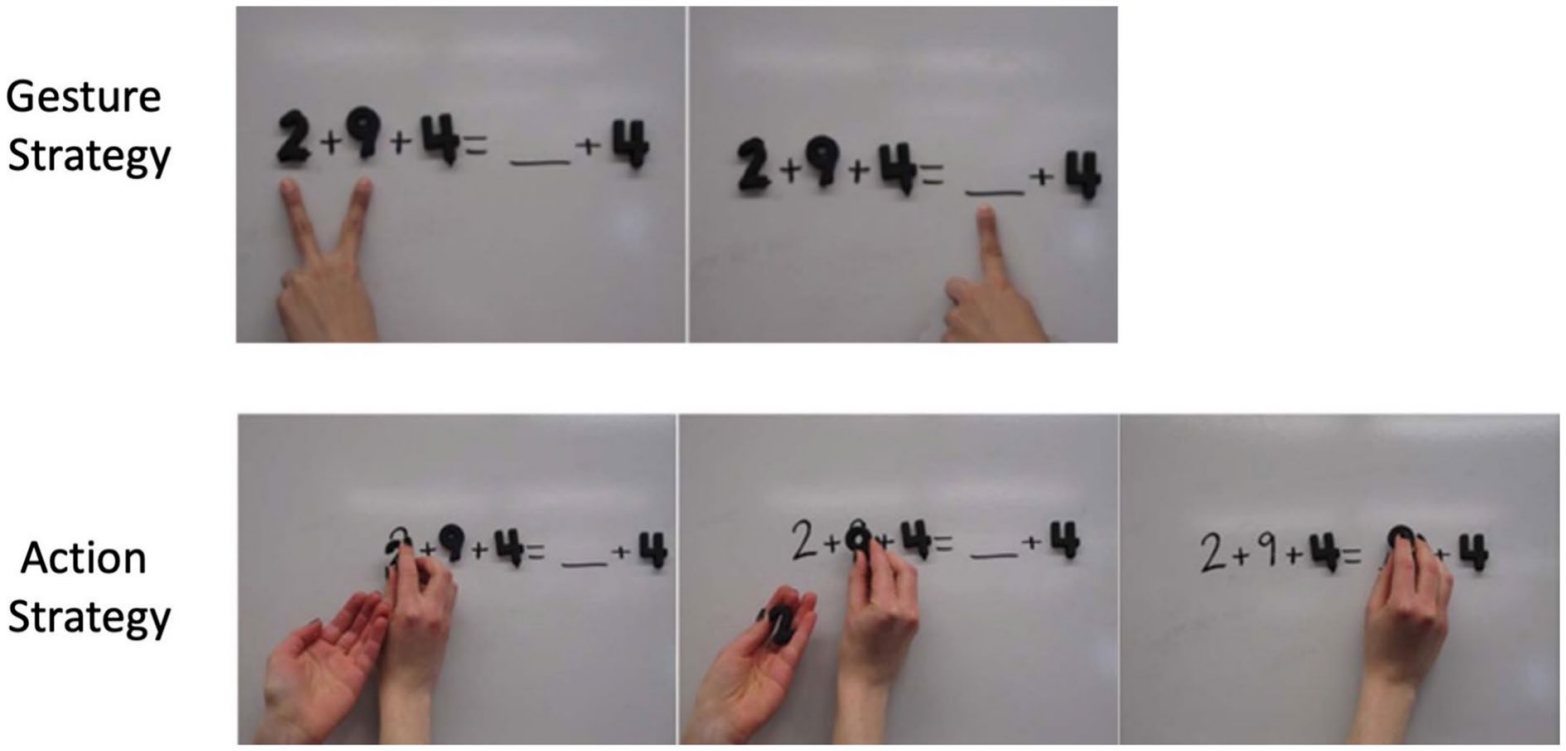
Demonstration of gestures (top row) and actions (bottom row) that students were taught to produce during instruction. Both movements convey the ‘grouping strategy;’ the correct answer for problems in this format (Form A) can be determined by adding or “grouping” the first two addends on the left side of the equation.

**Pre-Training and Training**. After completing their pre-test explanations, children received two blocks of one-on-one instruction describing how to correctly solve the missing addend equivalence problems. This instruction was based most directly on conditions designed by Novack et al. (2014) to assess the impact of gesture versus action instruction on mathematical equivalence in a tightly controlled manner, and also built off of four decades of work in which researchers have investigated how gesture could be used to support mathematical equivalence understanding (see Wakefield and Goldin-Meadow, 2019). Each block followed a similar structure. First, as an introduction to the training (“pre-training”), children were taught to say words and produce hand movements (actions or gestures) that they would be asked to produce during training. During this pre-training, they learned the words and hand movements by practicing on two Form A problems with the experimenter. The experimenter did not provide the answers during the pre-training phase but did provide feedback on performing the movements if the child needed it. In both conditions (Action and Gesture), children were taught to say an equalizer strategy, “I want to make one side equal to the other side,” while performing their movement strategy, which varied by condition (Figure 2). Children in the gesture condition (*n* = 35) were taught to produce the grouping strategy in gesture (a V-handshape with the index finger and middle finger placed under the first two numbers of the problem, followed by a single index finger from the same hand placed under the blank on the right side of the problem). Children in the action condition (*n* = 36) were taught to produce the grouping strategy with actions on magnetic number tiles (children picked up the first two number tiles on the left side of the equation with one hand, placing each tile in their second hand, and then picked them back out of the second hand and held them together directly over the blank using their first hand). Prior work has found that producing (or observing) two different strategies, one in speech and another in gesture, improves learning over-and-above producing the same two strategies entirely in speech or producing only one of the strategies in both speech and gesture (e.g., Goldin-Meadow et al., 1999; Congdon et al., 2017; Carrazza et al., 2021).

During training, the experimenter and child took turns solving three different Form A problems each, for a total of 6 problems. When it was the experimenter’s turn, she wrote the correct answer in the blank and then stated the concept of equivalence in speech: “I want to make one side equal to the other side.” Next, the experimenter elaborated by verbally summing the numbers of each side to show how the two sides are equal, for example: “2 plus 9 plus 4 is 15 and 11 plus 4 is 15.” Finally, the experimenter repeated the concept of equivalence by saying, “so one side is equal to the other side.” The experimenter did not produce any hand movements during this phase. When it was the child’s turn, the experimenter wrote a new Form A equivalence problem on the board and asked the child to produce the speech and movement strategies learned during pre-training. The experimenter then asked the child to try solving the problem, and finally, asked the child to repeat the strategies one more time. The experimenter told the child whether their solution was correct or incorrect but did not provide the correct answer or any additional feedback. Block 2 then followed the same procedure. The experimenter repeated the pre-training phrase as an instructional reminder and modeled the same instruction strategy. The experimenter and child took turns solving an additional three Form A problems each, for a total of 12 Form A problems across the two blocks.

**Post-test**. After training, children completed a paper-and-pencil post-test that was identical in form to the pre-test but contained a different set of six problems, two each of Forms A, B, and C. During post-test, children were asked to solve the problems and were not instructed to repeat the speech and movement strategies they learned during training (though they were not stopped if they chose to do so).

### Testing days 2 and 3

Children completed two follow-up pencil-and-paper tests, 1 day and 4 weeks after Testing Day 1. Assessments were identical in format to the written pre-test and post-test and consisted of six new problems with all three problem types (Forms A, B, and C).

### Evaluating individual differences in spontaneous co-speech gestures

Children’s explanations of the four equal-addend pre-test problems (Form A and Form B problems) were coded for verbal and gesture strategies. Speech and gesture were first transcribed from video recordings, and then coded independently by four coders following the strategies described in Alibali and Goldin-Meadow (1993). Verbal strategies were coded by listening to the child’s spoken explanation of how they solved the problem. Gesture strategies were coded by observing the video without reference to the speech. Any initial disagreements between coders were discussed to arrive at a final code. If there was a disagreement about codes and a final code could not be agreed upon by all coders, a final code was given based on the majority of coders. Children who produced different strategies in speech and gesture on at least one of the pre-test problems were classified as *Mismatchers*.^2^ Children who did not produce different strategies in gesture and speech on any of the pre-test problems were classified as *Non-Mismatchers*. Children classified as non-mismatchers either conveyed the same strategy through speech and gesture, or produced an incomplete gesture strategy that was not different from the strategy they produced in speech. Children who did not gesture during their explanations on any of the four pre-test problems were classified as *No Gesturers* and excluded from the sub-group analysis of individual differences.

## Study 1: Results

All analyses were conducted in R (version 4.2.1, “Funny-Looking Kid”) and R-Studio (v2022.07.0 + 544, “Spotted Wakerobin”) using the glmer function in the lme4 package (version 1.1–30; Bates et al., 2015). All code is presented in Supplementary material 1. Data and code are available from the Open Science Framework.^3^

Our primary analysis consisted of a mixed-effects logistic regression model predicting accuracy (0 or 1) on each post-test problem. The regression included fixed effects of condition (action training or gesture training), post-test time (numerically coded using scaled codes of −1 for the immediate post-test, 0 for the 24-h delayed post-test, and 1 for the 4-week delayed post-test), problem type (Form A, B, or C), and their interactions. We also included a random effect of participant ID because each participant contributed multiple data points. Follow-up analyses for interactions included terms for the main effects in the original models and, when relevant, significant interaction terms (*p* < 0.05).

Secondary sub-group analyses focused only on children who were either classified as mismatchers or non-mismatchers. Children who did not gesture or whose gestures and speech were not codable were excluded from the sub-group analyses. We tested for interactions between children’s pre-test gesture sub-group (Mismatcher or Non-Mismatcher), their training condition, and post-test time in a mixed-effects logistic regression. We included an effect for problem type based on the results of the primary analyses, as well as a random effect of participant ID to control for multiple data points per participant. As described for the primary analysis, only significant interaction terms were retained in the follow-up analyses (*p* < 0.05).

### Primary analyses: how do actions and gestures influence learning across time?

**Figure 3:**
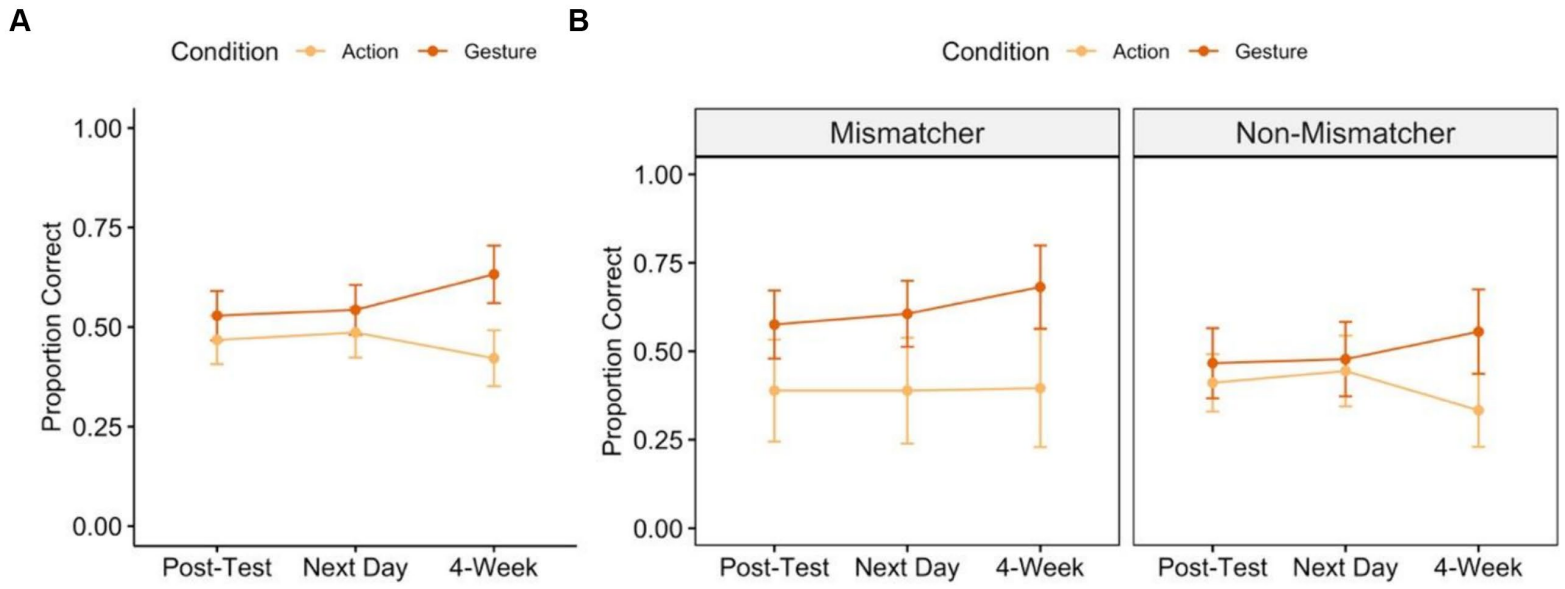
Post-test performance over time following action-based (light orange) and gesture-based (dark orange) training. Panel **A** shows average proportion correct across all children and Panel **B** shows average proportion correct for children with high implicit knowledge (“Mismatchers”) and low implicit knowledge (“Non-Mismatchers”). Error bars represent standard error of the mean of the participant-level data.

The primary analysis for Study 1 tested the prediction that gesture will boost retention of newly learned knowledge over-and-above action. If retention varies by training condition, we expect a significant interaction between condition and time. Figure 3A shows the average proportion correct at each post-test timepoint for children trained with action vs. gesture. Across both conditions and all timepoints, children showed significant improvement from pre-test to post-test [one-sample *t*-tests testing that proportion correct is greater than 0: immediate post-test: *Action:* mean proportion correct = 0.47, t(35) = 7.692, *p* < 0.0001; *Gesture:* mean = 0.53, t(35) = 8.518, *p* < 0.0001; next-day post-test: *Action:* mean = 0.49, t(35) = 7.701, *p* < 0.0001; *Gesture*: mean = 0.54 t(34) = 8.651, *p* < 0.0001; 4-week post-test: *Action*: mean = 0.42, t(33) = 5.984, *p* < 0.0001; *Gesture*: mean = 0.63, t(33) = 8.738, *p* < 0.0001].

A mixed-effects logistic regression revealed that there was a significant effect of problem type, and a significant interaction between condition and timepoint [main effects: *condition:* 𝛘^2^(1) = 0.693, *p* = 0.405; *time:* 𝛘^2^(1) = 1.561, *p* = 0.212; *problem type:* 𝛘^2^(2) = 142.763, *p* < 0.0001; interactions: *condition x time:* 𝛘^2^(1) = 9.087, *p* = 0.003; *condition x problem type*: 𝛘^2^(2) = 1.806, *p* = 0.405; *time x problem type:* 𝛘^2^(2) = 5.380, *p* = 0.068; *condition x time x problem type:* 𝛘^2^(2) = 0.756, *p* = 0.685].

The main effect of *problem type* demonstrated the predicted pattern: children performed significantly better on trained problem forms (Form A mean proportion correct = 0.70), relative to untrained problem forms (beta estimates from regression with Form A as the reference group: Form B: m = 0.42; *β* = −2.952, z = −8.279, *p* < 0.0001; Form C: m = 0.41; *β* = −2.788, z = −7.968, *p* < 0.0001). Repeating the regression with Form B as the reference group further revealed that there was no difference between performance on Form B and C problem types (Form C: *β* = 0.164, *z* = 0.519, *p* = 0.604). The absence of an interaction between *problem type and condition* suggests that this pattern was consistent across both training conditions (see Supplementary material 2 for a breakdown of these patterns within each condition). Importantly, performance for all three problem types was significantly above 0 [one-sample t-tests testing that proportion correct is greater than 0: Form A: t(70) = 13.986, *p* < 0.0001; Form B: t(70) = 8.299, *p* < 0.0001, Form C: t(70) = 8.801, *p* < 0.0001], demonstrating that children showed significant improvement from pre-test, not only on trained problems, but also on the novel forms.

To unpack the interaction between *time and condition* we performed separate analyses on the effect of time within each condition, controlling for problem type. Interestingly, performance *increased* over time in the gesture condition, but not in the action condition [Action Training: *time:* 𝛘^2^(1) = 1.831, *p* = 0.176; *problem type:* 𝛘^2^(2) = 80.510, *p* < 0.0001; Gesture Training: *time:* 𝛘^2^(1) = 11.231, *p* = 0.0008; *problem type:* 𝛘^2^(2) = 67.371, *p* < 0.0001]. In other words, children who trained through gesture showed an increase in correct problem-solving in the month following training, whereas children who trained through action showed consistent problem-solving.

To further understand how gesture and action support learning, we considered the pattern of errors made across all three post-test timepoints. Based on Novack et al. (2014), we would expect that children who learned through action would make a particular type of error more often: incorrectly adding together the first two addends and putting that sum in the blank on Form B and Form C problems. This type of response would reflect a more rigid understanding of equivalence, based on simply applying the algorithm shown in the action strategy to all problems (i.e., add the first two addends together). In contrast, Novack et al. (2014) found that children learning through gesture showed a more flexible understanding of the concept of equivalence and were able to correctly solve problems in a variety of formats. Focusing on errors made on Form B and C problems, we find that a higher proportion of adding-the-first-two-numbers errors were made by children in the action condition (means across all timepoints: Form B: 0.40; Form C: 0.43) than by children in the gesture condition (means across all timepoints: Form B: 0.33, Form C: 0.32; see Table 1 for a breakdown by post-test time). A mixed effect logistic regression, predicting the likelihood of making this type of error (1, 0) by condition, controlling for time point and problem type revealed that this difference was marginally significant: Children in the action condition made more adding-the-first-two-numbers errors than children in the gesture condition [χ^2^(1) = 3.50, *p* = 0.062]. This pattern suggests that children who learned through action showed a less flexible understanding of mathematical equivalence than children who learned through gesture, which conceptually replicates the findings from Novack et al. (2014).

**Table 1:** Proportion of incorrect post-test answers with an add-the-first-two-numbers answer.

	Immediate post-test		24-h post-test		4-Week post-test	
	Form B	Form C	Form B	Form C	Form B	Form C
Study 1						
Action	0.51	0.58	0.41	0.33	0.27	0.36
Gesture	0.37	0.47	0.36	0.23	0.24	0.23
Study 2						
Action→Gesture	0.41	0.59	0.42	0.34	0.29	0.33
Gesture→Action	0.40	0.45	0.40	0.28	0.14	0.11

### Secondary analyses: individual differences at pre-test

In a secondary sub-group analysis, we explored whether gesture had a greater effect on retention than action for participants categorized according to their pre-test understanding of mathematical equivalence (children who produced gesture-speech mismatches were assumed to have more implicit knowledge about mathematical equivalence and, in fact, produced more different problem-solving strategies at pre-test than children who did not produce mismatches). We found that 19 children (N_Action_ = 9, N_Gesture_ = 11) were classified as *mismatchers* and 30 were classified as *non-mismatchers* (N_Action_ = 15, N_Gesture_ = 15). Twenty children were excluded from these follow up analyses either because they produced no pretest gesture (N_Action_ = 11, N_Gesture_ = 9) or because they had a missing video (N _Action_ = 1).

Replicating Goldin-Meadow et al. (1993a), we found that children classified as mismatchers conveyed an average of 3.50 (SD = 0.95) unique strategies across speech and gesture, significantly more than non-mismatchers, who conveyed an average of 2.00 (SD = 0.69) unique strategies [*F*(1,48) = 41.806, *p* < 0.001]. The two groups were similar in their strategy use in speech [mismatchers = 1.55 different spoken strategies, SD = 0.60; non-mismatchers = 1.57, SD = 0.57, F(1,48) = 0.010, *p* = 0.922], indicating that the difference between the two groups lay in gesture [mismatchers = 3.05 different gesture strategies, SD = 0.76; non-mismatchers = 1.33, SD = 0.55, F(1,48) = 86.531, *p* < 0.001].

Next, we tested for interactions between children’s pre-test gesture sub-group (mismatcher vs. non-mismatcher), training condition (action, gesture), and post-test time point (immediate, 24-h delay, one-week delay), also including the fixed-effect of problem type that emerged from the primary analysis (Figure 3B). We found that pre-test gesture sub-group did *not* interact with condition or time and that the interaction between condition and time reported above persisted, even when controlling for mismatcher status [main effects: *pre-test gesture sub-group:* 𝛘^2^(1) = 0.039, *p* = 0.844; *condition:* 𝛘^2^(1) = 1.304, *p* = 0.253; *time:* 𝛘^2^(1) = 2.324, *p* = 0.127; *problem type:* 𝛘^2^(2) = 97.715, *p* < 0.0001; interactions: *pre-test gesture sub-group x condition:* 𝛘^2^(1) = 0.808, *p* = 0.369; *pre-test gesture sub-group x time*: 𝛘^2^(1) = 0.019, *p* = 0.891; *condition x time:* 𝛘^2^(1) = 6.867, *p* = 0.009; *pre-test gesture sub-group x condition x time:* 𝛘^2^(1) = 0.021, *p* = 0.885]. This pattern suggests that, regardless of a child’s pre-test status as a mismatcher, gesture-based training leads to an increase in problem-solving performance over time; action-based training does not (Figure 3B).

## Interim discussion

We found that, overall, children showed an increase in problem-solving performance over a four-week period following gesture training, but did not show a parallel increase in performance over time after action training. What is particularly striking is that the children in the gesture condition continued to improve *after the lesson* and made significant gains by the 4-week mark. This finding is not only consistent with previous work showing that gesture-based training in mathematical equivalence promotes better retention over time than speech-alone training (Cook et al., 2008, 2013; Congdon et al., 2017), but also parallels findings from previous work comparing gesture- and action-based instruction in a mental rotation task––gesture-based training led to more improvement after the lesson ended than action-based training (Levine et al., 2018). Similarly, the results of our error analysis are consistent with previous work by Novack et al. (2014), who showed that children learning to solve equivalence through an action strategy developed a more rigid, surface-level understanding than those who learned through gesture.

Why do children trained with gesture continue to show improvement even after the lesson is over and build a deeper conceptual understanding of equivalence? Desirable difficulty is one possibility. According to the desirable difficulty framework (Bjork, 1994; see also the optimal challenge point framework, Guadagnoli and Lee, 2004), training that engages people in deeper processing may slow down learning in the short run but result in more learning gains over time (Vlach et al., 2012; Szpunar et al., 2013). The gesture-based instruction condition in our study may have offered just the right level of difficulty to promote slow, but continuing, learning. In addition, gesture provided a more abstract representation of the grouping strategy, which led to more flexible learning.

Our secondary sub-group analysis revealed that gesture-based training was more effective than action-based training whether or not children had implicit knowledge of equivalence on the pre-test. Recall that children who produced gesture-speech mismatches also produced more different types of problem-solving strategies prior to training and, in this sense, were more prepared to learn equivalence than children who did not produce mismatches. Previous work has found differences between learning from action- and gesture-training based on a learner’s starting state knowledge, but in a different age group (1st graders) and a different task (measurement). Congdon et al. (2018) compared action- and gesture-based instruction in how to use a ruler to measure objects, and found that children who used a less sophisticated, incorrect, strategy benefited more from action-based instruction than gesture-based instruction; children who used a more sophisticated, yet still incorrect, strategy benefited equally from action and gesture.

Action-based instruction has been found to work particularly well with young children (see, for example, Novack et al., 2015, 2018), which could explain this difference between the measurement study and ours. However, there were other differences between the studies. For example, the movements used in both action and gesture training in the measurement study demonstrate how to count the intervals between the hatches on the ruler to determine the length of an object. In contrast, the movements used in our study instantiated a grouping problem-solving strategy and, as such, represented abstract transformations of the numbers in the problem. The movements used to teach mathematical equivalence thus seem to require a bigger conceptual leap than the movements used to teach measurement. Action may be a better teaching tool than gesture for beginners when the movements used to teach directly embody the problem-solving strategy.

In Study 2, we combine action- and gesture-based instruction within a single math equivalence lesson and vary the order of the two types of instruction. We explore movements that represent the abstract transformations of numbers and ask whether action can be made more effective as a teaching tool if it precedes gesture training than if it follows it. Based on Congdon et al. (2018), we speculate that learning about mathematical equivalence initially through action may help beginners make better use of gesture later.

### Study 2

In Study 1, we found that gesture promotes retention of knowledge gained during instruction better than action in all children, regardless of their knowledge status at pre-test. Study 2 uses the same paradigm to explore a mechanistic question that arises from Study 1—might gesture and action, two similar but distinct forms of movement, work well together in instruction, complementing one another because one is more concrete and the other more abstract?

Researchers and educators have established that learning occurs best when new concepts are introduced in stages that begin with the concrete and then transition to the abstract (e.g., Piaget, 1953; Bruner, 1966; Fyfe et al., 2014; Congdon et al., 2018). Bruner (1966) outlined a trajectory with three stages wherein the first stage uses enactive forms, which are physical and concrete models; the next stage uses iconic forms, which are graphic or pictorial models; and the final stage uses symbolic forms like language or numbers, which are the most abstract models. More recently, this description of the learning process has been coined ‘concreteness fading’ because there is good evidence that individuals learn best when content progresses from the most concrete to the least concrete models. Concreteness fading has been identified in several domains (Lesh, 1979; Gravemeijer, 2002; Lehrer and Schauble, 2002; Goldstone and Son, 2005; Fyfe et al., 2014) and has been used to teach children mathematical equivalence (Fyfe et al., 2015).

The action-based instruction used in Study 1 is concrete in the sense that its movements involve physical interaction with the world, leading to measurable changes in the environment. Conversely, the gesture-based instruction is abstract in that the hand movements do not result in physical movement of the number tiles but represent a strategy that can be performed on the numbers. If concrete training is best when it precedes abstract training, then instruction that begins with action and transitions to gesture should be more effective than instruction that moves in the reverse direction. We might expect this effect to be particularly pronounced for children with the lowest levels of prior knowledge about the task, as previous work has found that these children may be confused by being asked to perform an unfamiliar gesture (Congdon et al., 2018). As in Study 1, we classify children Study 2 who do *not* produce mismatches prior to instruction as having less prior knowledge about equivalence than children who do produce mismatches. Recall that children in Study 1 produced *fewer* different problem-solving strategies on the pre-test than children who produced mismatches, as has been found in previous work (Goldin-Meadow et al., 1993a).

## Study 2: Methods

### Participants

For Study 2 we worked with 71 third- and fourth-grade students (*M* = 9.4 years, SD = 0.6 years; 40 females) who did not correctly solve any pre-test problems and had not participated in Study 1. As in Study 1, participants were recruited from public elementary 3rd and 4th grade classrooms and were racially, ethnically, and socioeconomically diverse (Race Parent Report: 29.6% White, 4.2% Asian, 1.4% Black, 7% More than one race, 0% Native Pacific Islander, 4.2% Native American, 53.5% Unknown/Unreported; Ethnicity Parent Report: 56.3% Hispanic, 23.9% Not Hispanic, 19.7% Unknown/Unreported) and came from lower SES households: 52.4% of parents reported having a high-school degree or less; 19.7% reported having some college experience; 27.9% reported having a Bachelor’s or graduate degree (14.1% Unknown/Unreported). Prior to the study, parents provided written consent and children gave assent. Children received a small prize and certificate of participation, and teachers of participating classrooms received a gift card to a local learning store.

### Design and procedure

The design and procedure of Study 2 were identical to Study 1, except that all children were taught to produce strategies in action and in gesture. Children were randomly assigned to one of two instruction conditions: action followed by gesture (AG; *n* = 36) or gesture followed by action (GA; *n* = 35).

In Block 1 of training, children performed their first assigned movements. As in Study 1, they practiced their movements on two Form A problems in the pre-training phase and then took turns with the experimenter, each solving 3 Form A problems in the training phase. In Block 2, children were taught the other movement strategy during the pre-training phase of this block. They then took turns with the experimenter solving an additional 3 Form A problems each using the second movement strategy. As in Study 1, children completed 3 days of testing, and all assessments included 2 problems each of Form A, B, and C (see Figure 1).

### Evaluating individual differences in spontaneous co-speech gestures

As in Study 1, children’s pre-test explanations were coded for their verbal and gesture strategies and children were classified based on the criteria used in Study 1.

## Results

Analyses were conducted as described in Study 1. Data from all children were included in the mixed effects logistic regression in the primary analyses, but only data from children who were categorized as non-mismatchers or mismatchers were included in the secondary sub-group analyses. The two levels for condition in these analyses were action training followed by gesture training (AG training), and gesture training followed by action training (GA training).

### Primary analysis: does the order of instruction affect learning outcomes?

The primary analysis tested the prediction that action training followed by gesture training (AG training) would lead to better learning than gesture training followed by action training (GA training). If AG instruction leads to better learning overall, we should find a main effect of condition such that children who were trained with action first (AG) perform better across the post-tests than children who were trained with gesture first (GA). Another possibility is that AG training leads to better retention *over time* than GA training, similar to the pattern of results reported in Study 1. In that case, we would expect an interaction between condition and time, perhaps showing that children in the AG condition perform better than children in the GA condition, but only at the one-month delayed time point.

**Figure 4:**
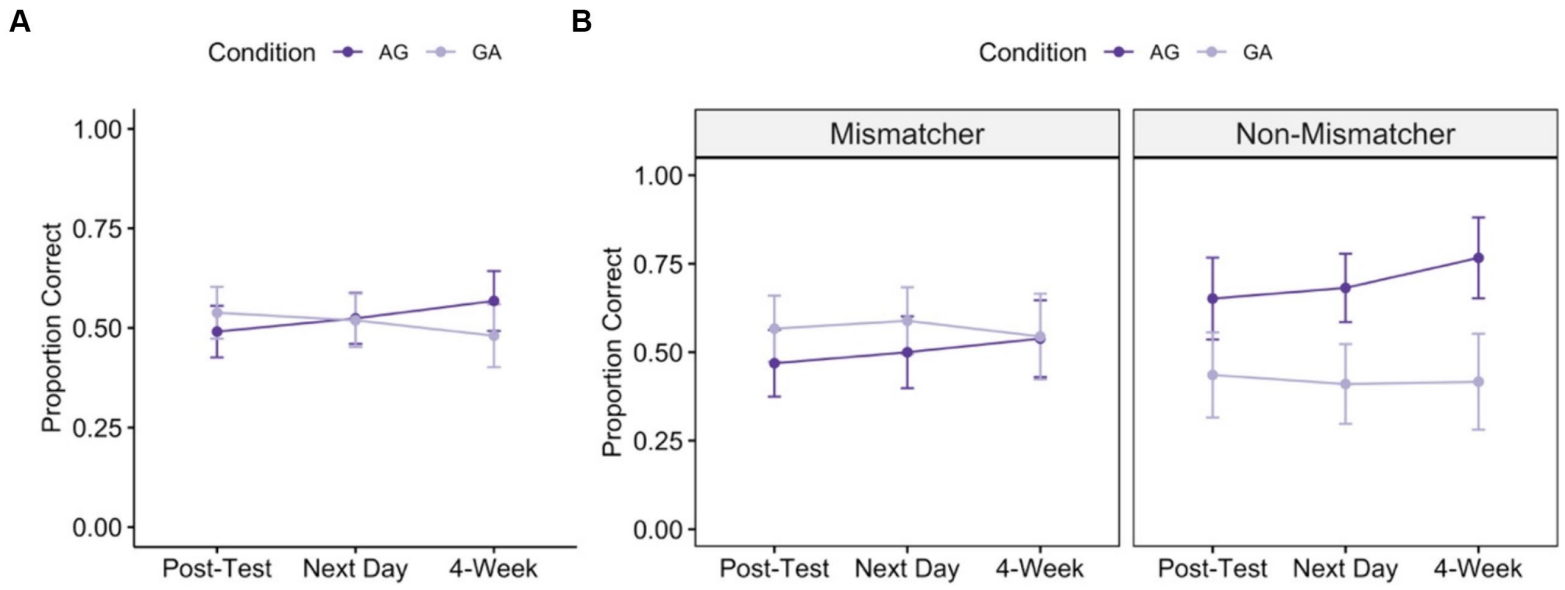
Post-test performance over time following instruction with action-then-gesture training (AG, dark purple) and gesture-then-action training (GA, light purple) training. Panel **A** shows average proportion correct across all children and Panel **B** shows average proportion correct for children with high implicit knowledge (“Mismatchers”) and low implicit knowledge (“Non-Mismatchers”). Error bars represent standard error of the mean of the participant-level data.

Figure 4A shows the average proportion correct at each timepoint for GA training and AG training. Overall, as in Study 1, children showed clear learning from the instruction. They show significant improvement from pre-test at all timepoints, with a slight upward trend in performance across time for children trained through AG instruction, and a slight downward trend in performance across time for children trained through GA instruction [see Figure 4A; one-sample t-tests testing that proportion correct is greater than 0: immediate post-test: *AG*: mean proportion correct = 0.49, t(35) = 7.585, *p* < 0.0001; *GA*: m = 0.54, t(34) = 8.297, *p* < 0.0001; next-day post-test: *AG*: m = 0.52, t(34) = 8.166, *p* < 0.0001; *GA*: m = 0.52, t(34) = 7.762, *p* < 0.0001; 4-weeks: *AG*: m = 0.57, t(31) = 7.554, *p* < 0.0001; *GA*: m = 0.48, t(33) = 6.088, *p* < 0.0001].

A mixed-effects logistic regression revealed no main effect of condition, but a significant main effect of problem type, and significant interactions between time and problem type, and between time and condition [main effects: *condition:* 𝛘^2^(1) = 0.085, *p* = 0.771; *time:* 𝛘^2^(1) = 1.081, *p* = 0.298; *problem type:* 𝛘^2^(2) = 92.349, *p* < 0.0001; interactions: *condition x time:* 𝛘^2^(1) = 12.377, *p* = 0.0004; *condition x problem type*: 𝛘^2^(2) = 2.092, *p* = 0.351; *time x problem type:* 𝛘^2^(2) = 24.814, *p* < 0.0001; *condition x time x problem type:* 𝛘^2^(2) = 0.751, *p* = 0.687].

To follow up on the interaction between *time and condition*, we looked at patterns of learning within each condition across the three time points. Children who were trained with action followed by gesture (AG) showed a significant increase in performance across the 4-week period, whereas children who were trained with gesture followed by action (GA) showed no significant change in performance across the time [Action-then-Gesture Training: *time:* 𝛘^2^(1) = 9.048, *p* = 0.003; *problem type:* 𝛘^2^(2) = 57.228, *p* < 0.0001; *time x problem type:* 𝛘^2^(2) = 12.286, *p* = 0.002; Gesture-then-Action Training: *time:* 𝛘^2^(1) = 1.887, *p* = 0.170; *problem type:* 𝛘^2^(2) = 37.591, *p* < 0.0001; *time x problem type:* 𝛘^2^(2) = 13.284, *p* = 0.001].

The interaction between time and problem type indicated that, although children continued to perform best on trained problems across all 3 time points, children performed better on Form C problems than Form B problems at the 24-h delayed post-test, but not at the immediate or 4-week delayed post-tests (statistics available in Supplementary material 2). Importantly, performance on all three types of problem types improved significantly from pre-test across all three time points [Immediate Post-Test: *Form A*: m = 0.73, t(70) = 14.525, *p* < 0.0001; *Form B*: m = 0.39, t(70) = 7.071, *p* < 0.0001; *Form C*: m = 0.42, t(70) = 7.488, *p* < 0.0001; 24-h Delayed Post-Test: *Form A*: m = 0.69, t(69) = 12.698, *p* < 0.0001; *Form B*: m = 0.39, t(69) = 6.790, *p* < 0.0001; *Form C*: m = 0.49, t(69) = 9.994, *p* < 0.0001; 4-Week Delayed Post-Test: *Form A*: m = 0.59, t(65) = 9.845, *p* < 0.0001; *Form B*: m = 0.50, t(65) = 8.318, *p* < 0.0001; *Form C*: m = 0.48, t(65) = 8.149, *p* < 0.0001].

As in Study 1, we considered whether the pattern of errors made could provide insight into what children took from instruction. Given that all children were exposed to action *and* gesture strategies, we might expect that the pattern of errors would look similar across the groups. We once again considered the proportion of errors made that reflected an add-the-first-two-numbers strategy: Children in the Action→Gesture condition made approximately the same proportion of these errors (mean across all timepoints: Form B: 0.38; Form C: 0.44) as children in the Gesture→Action condition (Form B: 0.32; Form C: 0.28; Table 1 for a breakdown by post-test time). A mixed effect logistic regression, predicting the likelihood of making an add-the-first-two-numbers error (1, 0) by condition, controlling for time point and problem type, revealed no significant difference between conditions [𝛘^2^(1) = 1.813, *p = 0*.179].

### Secondary analysis: individual differences at pre-test

As in Study 1, we also wanted to know whether children who had more or less knowledge of equivalence prior to instruction would differ in the impact that instruction had on their retention. In this sample, 30 children were then classified as *mismatchers* (AG = 16, GA = 15), and 24 were classified as *non-mismatchers* (AG = 11, GA = 13). Sixteen children were not included in this secondary analyses either because they did not produce gestures at pretest (AG = 7, GA = 7), or because their gestures were partially obstructed in the camera view so could not be assessed as either mismatchers or non-mismatchers.

As in Study 1, mismatchers conveyed more unique strategies across speech and gesture than non-mismatchers (mismatchers = 3.65, SD = 1.05, non-mismatchers = 2.46, SD = 0.66; *F*(1,53) = 23.46, *p* < 0.0001). The two groups were similar in their strategy use in speech (mismatchers = 1.68, SD = 0.75; non-mismatchers = 1.67, SD = 0.70, *F*(1,53) = 0.003, *p* = 0.957) but distinct in their use of unique strategies expressed in gesture (mismatchers = 3.00, SD = 1.21; non-mismatchers = 1.67, SD = 0.70, *F*(1,53) = 23.03, *p* < 0.001).

Next, we conducted our secondary analysis testing for interactions between children’s pre-test gesture sub-group (mismatcher vs. non-mismatcher), training condition, and time point. This model also included an interaction of time and problem type (to control for the effect of problem type found in the primary analysis). If children’s mismatch status predicts whether getting concrete action before abstract gesture is better for learning than the reverse order, we would expect an interaction between condition and pre-test gesture sub-group, or a three-way interaction between condition, time, and pre-test gesture sub-group.

Our regression model revealed the predicted two-way interaction between pre-test gesture sub-group and condition [main effects: *pre-test gesture sub-group:* 𝛘^2^(1) = 0.070, *p* = 0.792; *condition:* 𝛘^2^(1) = 1.331, *p* = 0.249; *time:* 𝛘^2^(1) = 3.025, *p* = 0.082; interactions: *pre-test gesture sub-group x condition:* 𝛘^2^(1) = 4.279, *p* = 0.039; *pre-test gesture sub-group x time*: 𝛘^2^(2) = 0.571, *p* = 0.450; *time x condition:* 𝛘^2^(1) = 4.584, *p* = 0.032; *time x problem type:* 𝛘^2^(2) = 21.662, *p* < 0.001; *pre-test gesture sub-group x condition x time:* 𝛘^2^(1) = 0.008, *p* = 0.929]. Follow-up analyses showed that, for non-mismatchers (children who expressed relatively *few* different problem-solving strategies prior to instruction and thus had comparatively lower prior knowledge), AG training resulted in significantly better post-test performance than GA training [main effects: *condition:* 𝛘^2^(1) = 3.950, *p* = 0.047; *time:* 𝛘^2^(1) = 2.954, *p* = 0.086; interactions: *condition x time*: 𝛘^2^(1) = 1.776, *p* = 0.183; *time x problem type:* 𝛘^2^(2) = 2.535, *p* = 0.282]. In contrast, mismatchers (children who expressed relatively *more* different problem-solving strategies prior to instruction, i.e., children with comparatively higher prior knowledge) showed no differences based on the order in which they received action and gesture training [main effects: *condition:* 𝛘^2^(1) = 0.236, *p* = 0.627; *time:* 𝛘^2^(1) = 0.752, *p* = 0.386; interactions: *condition x time*: 𝛘^2^(1) = 2.556, *p* = 0.110; *time x problem type:* 𝛘^2^(2) = 21.214, *p* < 0.001].^4^ These patterns are depicted in Figure 4B.

## Interim discussion

In Study 2, we gave all children a combination of action and gesture instruction but varied the order in which that instruction was received. Children learned from both conditions, suggesting that combining gesture and action within a single lesson is an effective approach for teaching math equivalence. They also made a similar number of add-the-first-two-numbers errors, suggesting that presenting gesture instruction along with action instruction helped children move away from simply applying the algorithm shown in the movement strategy when solving problems. However, we found that the optimal order in which to combine gesture and action training within a single lesson varied significantly based on their knowledge of equivalence prior to instruction (as assessed by the speech and gesture children produced during their pre-test problem-solving explanations). For children who did *not* produce spontaneous speech-gesture mismatches prior to training, it was better to receive action-based training before gesture-based training. For children who *did* produce speech-gesture mismatches prior to training, the order of action and gesture training did not matter.

This pattern is consistent with previous work in math equivalence showing that progressing from a concrete instructional tool to an abstract instructional tool can promote children’s ability to solve missing addend problems (Fyfe et al., 2015). It is notable that, in prior instantiations of “concreteness fading,” the concrete teaching examples have tended to reflect concrete enactment (e.g., manipulatives placed on a balance scale) whereas in this study the “concrete action” involved manipulating symbols (i.e., number tiles). Future work could examine how providing other types of concrete action instruction (including actions that are even more enacted) prior to a gesture instruction further enhances learning.

However, in the current study, we found that this effect was specific to children who did not produce spontaneous speech-gesture mismatches prior to training. Recall that children who did not produce mismatches also produced fewer problem-solving strategies prior to instruction than their mismatching peers. We suggest that these children had a weaker understanding of the concept to start, and may have needed the scaffolding provided by action training in order to subsequently make good use of the gesture training. As a first step, the action training may have allowed learners to more concretely see how the addends should be combined to solve the problem. This understanding may then have helped them to better understand the representational function of the gesture, which allowed them to apply their knowledge to the post-tests.

In contrast, children who produced mismatches and produced comparatively more different problem-solving strategies prior to instruction initially had more ideas about how to solve the problems than children who did not produce mismatches (although many of the ideas were implicit and not expressed in speech). These children did not need the scaffolding from action in order to understand how to glean information from gesture and may not even have needed action at all––previous work has shown that techniques supporting learning for novices do not always work well for learners with more initial knowledge (Kalyuga, 2007; Homer and Plass, 2010).

## General discussion

Decades of research have shown that gesture is a powerful tool for learning, particularly in the domain of mathematics (Wakefield and Goldin-Meadow, 2019). Our goal in the two current studies was to further clarify the benefits, boundaries, and mechanisms of gesture-based instruction. To do so, we directly compared gesture-based instruction to action-based instruction, measuring learning over time, and exploring the impact of individual differences in knowledge prior to instruction on learning over time. We examined the effectiveness of gesture- and action-based instruction in two contexts: action- *or* gesture-based training over time (Study 1), and action- *and* gesture-based training over time (Study 2).

In Study 1, we found that gesture was better than action at promoting retention of math equivalence over time; 4 weeks after instruction, children in the gesture group were correctly solving significantly more missing-addend problems than children in the action group. We also found that children in the action condition were more likely to make add-the-first-two-number errors than children in the gesture condition, suggesting more flexible understanding of equivalence after gesture instruction.

In Study 2, we asked whether providing children with both types of movement instruction is effective for learning and, if so, whether the order of instruction matters. We found that children taught to produce action before gesture during instruction showed a significant increase in the number of problems they solved correctly over time. In contrast, children taught to produce gesture first showed consistent performance over time. Importantly, these effects were seen for both trained items and transfer items, and there was no difference in the proportion of add-the-first-two-number errors made across conditions, suggesting that the learning intervention did not just teach children a procedure, but rather boosted their conceptual understanding of equivalence.

Secondary sub-group analyses in each study evaluated whether individual differences in the mathematical equivalence knowledge children brought to instruction (as assessed by spontaneous co-speech gestures produced at pre-test) interacted with the patterns just reported. In Study 1, gesture promoted retention better than action for *all* children, regardless of their level of pre-test knowledge. In contrast, in Study 2, the orders in which the two types of instruction were presented had different effects on children as a function of their pretest knowledge Children who produced *no* mismatches at pre-test (and produced relatively few different types of problem-solving strategies before instruction) were not well prepared for the lesson. They displayed a significant increase in the number of problems solved correctly over time when taught to produce action before gesture, but not when taught to produce gesture before action. In contrast, children who *did* produce mismatches at pre-test (and produced relatively more different types of problem-solving strategies before instruction) performed the same no matter what order of instruction they received.

Previous work (Church and Goldin-Meadow, 1986; Perry et al., 1988) found that children who did not know how to solve a problem were more prepared to profit from instruction in the problem if their gestures and speech conveyed different information than if the two modalities conveyed the same information. Subsequent work has confirmed that spontaneous gesture in speakers (Alibali and Goldin-Meadow, 1993; Pine et al., 2004; Ping et al., 2021) and in signers (Goldin-Meadow et al., 2012) can be used to predict who is particularly likely to make progress on a task when given instruction. Our studies did not find a main effect of the children’s spontaneous co-speech gestures on learning and thus did not replicate previous findings. However, the instructional conditions we used here focused on how action and gesture impact learning and, in so doing, varied substantially from the instructional procedures used in previous work. For example, instruction in some previous studies used *only* speech *or* a movement strategy. Ping et al. (2021) taught college students about stereoisomers through speech only instruction, and Pine et al. (2004) taught children about balance through a demonstration with actual beams *without* spoken instruction. It may be that having two consistent routes by which to learn led our instruction to similarly benefit mismatchers and non-mismatchers. Nevertheless, in Study 2, we found that non-mismatchers made more learning gains following AG training than GA training, suggesting that non-mismatchers need concrete instruction before abstract instruction in order to learn, which is consistent with the hypothesis that they are *less* ready to learn mathematical equivalence than mismatchers (recall that mismatchers made progress on the task no matter what order the instructions was delivered in).

The current work raises important considerations for future work designed to investigate co-speech gesture as an individual difference measure of knowledge prior to instruction. For one, we chose a definition of ‘mismatcher’ that included children who produced *at least one* mismatch at pre-test. This decision was made partially because our study provided limited opportunities for children to produce mismatches. Future work could provide children with more opportunities to produce gesture-speech mismatches prior to instruction, and then consider whether more nuanced levels of pre-test mismatch predict learning better than a dichotomous split (see Perry et al., 1988, Goldin-Meadow et al., 2012, for evidence for the more nuanced measure). In addition, measuring children’s knowledge through their speech and gestures not only before, but also after, instruction could provide key insights into understanding how gesture and action promote learning.

Additionally, some learners did *not* spontaneously gesture at all when explaining their solutions to the math problems; there was thus no information about implicit strategies reflected in gesture for these children (see Garber et al., 1998, for evidence that gesture captures implicit knowledge). These learners might be qualitatively different in terms of learning potential from children who produce *some* spontaneous gesture at pre-test (Congdon et al., 2024). In future work, we suggest collecting larger samples to explore the types of input that are most effective for children who do and do not gesture, and for children whose gestures do and do not match their speech. We also suggest comparing gesture-speech mismatch to a second pre-test measure of mathematical equivalence to further validate mismatch as a measure of implicit pre-test knowledge.

In the current studies, we examined children’s learning following their own production of experimenter-dictated gesture and action strategies. One open question is whether action and gesture function similarly when *teachers* are the ones who act on objects or gesture. Seeing a teacher or instructor gesture can result in different learning outcomes, compared to when learners are instructed to gesture themselves. For example, when gesture is produced by the teacher, it is a more effective teaching tool if it is produced simultaneously with speech than if it is produced sequentially after speech (Congdon et al., 2017). But when gesture is produced by the learner, the timing of speech and gesture does not matter––sequential presentation is as effective as simultaneous presentation (Carrazza et al., 2021). These findings point to an important difference in how teacher gesture vs. student gesture affects learning.

In thinking about children’s experiences doing and seeing actions and gestures, it is worth considering whether our Study 2 findings can be explained, in part, by children’s familiarity with performing actions with manipulatives in classrooms. We know from previous work that teachers use gesture prolifically when explaining math concepts in classrooms (Neill and Caswell, 2003; Alibali and Nathan, 2012) and that children not only attend to their teacher’s gestures, but also learn and retain new knowledge from observing those gestures (Congdon et al., 2017; Wakefield et al., 2018b). Students might be more used to gleaning information from their teacher’s gestures than to being taught to produce specific gestures themselves during a lesson. By contrast, we know that teachers not only incorporate actions on manipulatives into their math lessons (Mix, 2010; Roschelle et al., 2010), but also encourage children to produce their own actions on manipulatives in the classroom (Lillard, 2005; Lillard and Else-Quest, 2006). Popular approaches, such as Montessori education, emphasize children’s own spontaneous exploratory actions (as well as teacher demonstrations) as a key component of the learning process, suggesting that children may be just as likely, or even more likely, to act on objects in the classroom, compared to teachers. The frequency of teachers’ and children’s gestures and actions in math classrooms may therefore differ and should be examined to determine whether common classroom practices align with (and perhaps explain) some of our experimental results better than the explanation we have proposed about the concreteness or abstractness of the movements themselves.

A related question is whether gesture’s benefits are the same across educational domains, and whether gesture’s impact on learning, especially relative to action, depends on the specific content of the to-be-learned-task. Certain topics, for example, learning about torque and angular momentum, lend themselves particularly well to an action-intervention, at least relative to passive observation (Kontra et al., 2015). It is an open question as to whether the impact of gesture instruction relative to action instruction is the same in these domains, or whether the generalization and retention boosts of gesture relative to action are specific to topics that have fewer physical connections.

Furthermore, there are many different ways that gesture and particularly action can be used to teach mathematical equivalence beyond the approach taken in our study. We did not design this study to develop the best way to teach this concept to children, and it may be that the movements used (i.e., picking up or gesturing toward numerical tiles and grouping them together) required a big conceptual leap to connect those movements to the concept. Alternative approaches could be developed that might better support learning. For example, using discrete, non-symbolic manipulatives (e.g., beads or base 10 blocks) that can be grouped together might provide children with a more transparent instantiation of the grouping strategy in action than the one used here. However, whether students combine symbols (that need to be added together to get the answer) or beads (that need to be counted together to get the answer), they still need to grasp the link between the symbols/beads and the equation, which is often the stumbling block in learning mathematical concepts. Future work needs to directly compare different ways of instantiating strategies in gesture and action to assess their impact on learning.

In sum, we have found that, regardless of individual differences in implicit knowledge of mathematical equivalence prior to instruction, instruction where learners use gesture on its own promotes retention over and above instruction where learners use action on its own. But when the two tools are combined, individual differences do seem to have an impact on learning. Children who have relatively few different types of problem-solving strategies in their mathematical equivalence repertoires prior to instruction (i.e., they produce no mismatches) profit from doing action before doing gesture during the lesson (and not the other way around). Children who have many different problem-solving strategies in their repertoires prior to instruction (i.e., they produce mismatches) benefit equally from doing action or doing gesture first.

Taken together, our findings indicate that gesture is a teaching device that not only promotes immediate learning, but more importantly, leads to retention (Study 1)––more powerful than comparable actions that are performed on objects. But action on objects can serve as a key concrete stepping-stone for children who are less prepared, allowing them to subsequently benefit from gesture (Study 2). Beyond the individual differences we have identified, it is important to emphasize the practical educational implications of our findings––gesture is a powerful tool, accessible to most learners at some point in the learning process, that leads to long-lasting and flexible understanding of challenging mathematical concepts.

## Data availability statement

Data and analysis code are available from the Open Science Framework (OSF) at: https://osf.io/dygr4/?view_only=762a7e43f7c64c22b6676eca5eb125fe.

## Ethics statement

The studies involving humans were approved by University of Chicago Institutional Review Board. The studies were conducted in accordance with the local legislation and institutional requirements. Written informed consent for participation in this study was provided by the participants’ legal guardians/next of kin.

## Author contributions

AK: Conceptualization, Data curation, Formal analysis, Methodology, Visualization, Writing – original draft. CC: Conceptualization, Formal analysis, Methodology, Visualization, Writing – original draft. MN: Conceptualization, Methodology, Writing – review & editing. EC: Conceptualization, Methodology, Writing – review & editing. EW: Conceptualization, Methodology, Writing – review & editing. NH-L: Investigation, Writing – review & editing, Supervision, Project administration. SG-M: Conceptualization, Funding acquisition, Resources, Supervision, Writing – review & editing, Methodology.

## Footnotes

^1^Across both Study 1 and Study 2, children who correctly solved any pre-test problems (*N* = 53, approximately 27% of the original sample) were excluded from participating and were not assigned to a training condition. Additionally, some children were excluded from particular analyses, and this is noted throughout the paper.

^2^The decision to classify mismatchers as children producing at least one mismatch differs from some prior studies that required 3 out of 6 pretest mismatches to be classified as a mismatcher. The pretest in the current study contained only 4 problems. As a result, children in the current study had fewer opportunities for to express mismatching strategies, compared to prior work.

^3^https://osf.io/dygr4/

^4^Direct comparisons of mismatchers and non-mismatchers for each training condition revealed no significant differences between pre-test gesture sub-group following either GA or AG training, though the effect was marginal for AG training [GA Training: *pre-test gesture subgroup:* 𝛘^2^(1) = 1.450, *p* = 0.228; *time:* 𝛘^2^(1) = 0.027, *p* = 0.869; *time x problem type:* 𝛘^2^(2) = 13.542, *p* = 0.001; AG Training: *pre-test gesture subgroup:* 𝛘^2^(1) = 3.108, *p* = 0.082; *time:* 𝛘^2^(1) = 6.884, *p* = 0.009; *time x problem type:* 𝛘^2^(2) = 8.964, *p* = 0.011]. This indicates that, as a group, children performed similarly on their post-tests regardless of whether or not they produced speech-gesture mismatches prior to instruction.
