# Supplementary material for: The effects of gesture and action training on the retention of math equivalence
Source: Front Psychol. 2024 Jul 4;15:1386187. doi: 10.3389/fpsyg.2024.1386187 (PMC11256877; doi:10.3389/fpsyg.2024.1386187)
Supplement: Supplementary file 1 [file Data_Sheet_1.PDF]

Supplement 1: Analyses for ‘The effects of gesture and action training on the retention of math equivalence’

06/21/2021

Study 1: Gesture vs Action Training

Post-test performance over time for action (light orange) and gesture (dark orange) instruction.

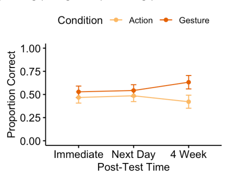

Condition	PostTestTime	meanProportionCorrect
Action	Immediate	0.468
Action	Next Day	0.486
Action	4 Week	0.422
Gesture	Immediate	0.529
Gesture	Next Day	0.543
Gesture	4 Week	0.632

Study 1 - Primary Analyses

Logistic Regression:

```
S1_Primary <-
  glmer(Correct ~ Condition*PostTestTime_Scaled*ProblemType + (1|SubID),
        data=S1,
        family=binomial, glmerControl(optimizer="bobyqa", optCtrl=list(maxfun=100000)))
```

	Chisq	Df	Pr(>Chisq)
Condition	0.694	1	0.405
PostTestTime_Scaled	1.561	1	0.212
ProblemType	142.767	2	0.000
Condition:PostTestTime_Scaled	9.087	1	0.003
Condition:ProblemType	1.806	2	0.405
PostTestTime_Scaled:ProblemType	5.380	2	0.068
Condition:PostTestTime_Scaled:ProblemType	0.756	2	0.685

Follow-Up Logistic Regressions:

Action Training over Time:

```
Act_TimeProbType <-
  glmer(Correct ~ PostTestTime_Scaled + ProblemType +  (1|SubID),
        data=subset(S1, Condition=="Action"),
        family=binomial, glmerControl(optimizer="bobyqa", optCtrl=list(maxfun=100000)))
```

	Chisq	Df	Pr(>Chisq)
PostTestTime_Scaled	1.831	1	0.176
ProblemType	80.510	2	0.000

	Estimate	Std. Error	z value	Pr(>|z|)
(Intercept)	1.640	0.644	2.548	0.011
PostTestTime_Scaled	-0.210	0.155	-1.353	0.176
ProblemTypeFormB	-2.887	0.353	-8.168	0.000
ProblemTypeFormC	-2.724	0.346	-7.866	0.000

Gesture Training over Time:

```
Gest_TimeProbType <-
  glmer(Correct ~ PostTestTime_Scaled + ProblemType +  (1|SubID),
        data=subset(S1, Condition=="Gesture"),
        family=binomial, glmerControl(optimizer="bobyqa", optCtrl=list(maxfun=100000)))
```

	Chisq	Df	Pr(>Chisq)
PostTestTime_Scaled	11.231	1	0.001
ProblemType	67.371	2	0.000

	Estimate	Std. Error	z value	Pr(>|z|)
(Intercept)	2.895	0.812	3.564	0.000
PostTestTime_Scaled	0.570	0.170	3.351	0.001
ProblemTypeFormB	-3.221	0.436	-7.395	0.000
ProblemTypeFormC	-3.526	0.445	-7.929	0.000

Immediate Post-Test, Action vs Gesture:

```
S1_T1<-glmer(Correct ~ Condition + ProblemType +  (1|SubID),
             data=subset(S1, PostTestTime=="Immediate"),
             family=binomial, glmerControl(optimizer="bobyqa", optCtrl=list(maxfun=100000)))
```

	Chisq	Df	Pr(>Chisq)
Condition	0.250	1	0.617
ProblemType	56.343	2	0.000

24-hr Delayed Post-Test, Action vs Gesture:

```
S1_T2<-glmer(Correct ~ Condition + ProblemType +  (1|SubID),
             data=subset(S1, PostTestTime=="Next Day"),
             family=binomial, glmerControl(optimizer="bobyqa", optCtrl=list(maxfun=100000)))
```

	Chisq	Df	Pr(>Chisq)
Condition	0.479	1	0.489
ProblemType	40.554	2	0.000

4-Week Delayed Post-Test, Action vs Gesture:

```
S1_T3 <- glmer(Correct ~ Condition + ProblemType +  (1|SubID),
               data=subset(S1, PostTestTime=="4 Week"),
               family=binomial, glmerControl(optimizer="bobyqa", optCtrl=list(maxfun=100000)))
```

	Chisq	Df	Pr(>Chisq)
Condition	3.158	1	0.076
ProblemType	24.621	2	0.000

Study 1 - Secondary Sub-Group Analyses

Study 1 Sub-Group Post-Test Performance over Time:

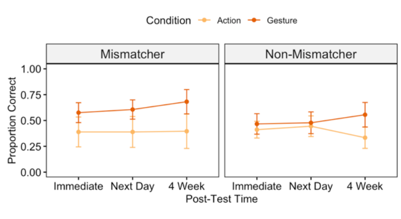

Study 1 Sub-Group Breakdown:

```
##
## ----------- ---------- ------------ ---------------- ------------- ----------- ------ -------
##                SubGroup   Mismatcher   Non-Mismatcher   No Gesturer   Uncodable   <NA>   Total
##   Condition
##      Action                        9               15            11           0      1      36
##     Gesture                       11               15             9           0      0      35
##       Total                       20               30            20           0      1      71
## ----------- ---------- ------------ ---------------- ------------- ----------- ------ -------
```

Logistic Regression:

```
S1_SubGrpReg <- glmer(Correct ~ SubGroup*Condition*PostTestTime_Scaled + ProblemType + (1|SubID),
                      data=S1sub, family=binomial,
                      glmerControl(optimizer="bobyqa", optCtrl=list(maxfun=100000)))
```

	Chisq	Df	Pr(>Chisq)
SubGroup	0.039	1	0.844
Condition	1.304	1	0.254
PostTestTime_Scaled	2.324	1	0.127
ProblemType	97.717	2	0.000
SubGroup:Condition	0.809	1	0.369
SubGroup:PostTestTime_Scaled	0.019	1	0.891
Condition:PostTestTime_Scaled	6.867	1	0.009
SubGroup:Condition:PostTestTime_Scaled	0.021	1	0.885

Follow-Up Logistic Regressions:

Action Training over Time controlling Pre-Test Gesture Sub-Group:

```
Act_TimeProbType_SubGrp <-
  glmer(Correct ~ SubGroup + PostTestTime_Scaled + ProblemType + (1|SubID),
        data=subset(S1sub, Condition=="Action"),
        family=binomial, glmerControl(optimizer="bobyqa", optCtrl=list(maxfun=100000)))
```

	Chisq	Df	Pr(>Chisq)
SubGroup	0.260	1	0.610
PostTestTime_Scaled	0.778	1	0.378
ProblemType	44.409	2	0.000

	Estimate	Std. Error	z value	Pr(>|z|)
(Intercept)	0.272	1.435	0.189	0.850
SubGroupNon-Mismatcher	0.901	1.767	0.510	0.610
PostTestTime_Scaled	-0.186	0.211	-0.882	0.378
ProblemTypeFormB	-2.795	0.478	-5.848	0.000
ProblemTypeFormC	-2.976	0.489	-6.079	0.000

Gesture Training over Time controlling Pre-Test Gesture Sub-Group:

```
Gest_TimeProbType_SubGrp <-
  glmer(Correct ~ SubGroup + PostTestTime_Scaled + ProblemType +(1|SubID),
        data=subset(S1sub, Condition=="Gesture"),
        family=binomial, glmerControl(optimizer="bobyqa", optCtrl=list(maxfun=100000)))
```

	Chisq	Df	Pr(>Chisq)
SubGroup	0.573	1	0.449
PostTestTime_Scaled	8.729	1	0.003
ProblemType	49.209	2	0.000

	Estimate	Std. Error	z value	Pr(>|z|)
(Intercept)	3.351	1.345	2.493	0.013
SubGroupNon-Mismatcher	-1.295	1.710	-0.757	0.449
PostTestTime_Scaled	0.568	0.192	2.954	0.003
ProblemTypeFormB	-3.248	0.522	-6.226	0.000
ProblemTypeFormC	-3.692	0.536	-6.893	0.000

Immediate Post-Test, Action vs Gesture controlling Pre-Test Gesture Sub-Group:

```
S1_T1_subgrp <-
  glmer(Correct ~ SubGroup*Condition + ProblemType +  (1|SubID),
        data=subset(S1sub, PostTestTime=="Immediate"),
        family=binomial, glmerControl(optimizer="bobyqa", optCtrl=list(maxfun=100000)))
```

	Chisq	Df	Pr(>Chisq)
SubGroup	0.114	1	0.736
Condition	0.854	1	0.355
ProblemType	37.705	2	0.000
SubGroup:Condition	0.586	1	0.444

24-hr Delayed Post-Test, Action vs Gesture controlling Pre-Test Gesture Sub-Group:

```
S1_T2_subgrp<-
  glmer(Correct ~ SubGroup*Condition + ProblemType +  (1|SubID),
        data=subset(S1sub, PostTestTime=="Next Day"),
        family=binomial, glmerControl(optimizer="bobyqa", optCtrl=list(maxfun=100000)))
```

	Chisq	Df	Pr(>Chisq)
SubGroup	0.101	1	0.750
Condition	0.973	1	0.324
ProblemType	26.986	2	0.000
SubGroup:Condition	0.733	1	0.392

4-week Delayed Post-Test, Action vs Gesture controlling Pre-Test Gesture Sub-Group:

```
S1_T3_subgrp <-
  glmer(Correct ~ SubGroup*Condition + ProblemType +  (1|SubID),
        data=subset(S1sub, PostTestTime=="4 Week"),
        family=binomial, glmerControl(optimizer="bobyqa", optCtrl=list(maxfun=100000)))
```

	Chisq	Df	Pr(>Chisq)
SubGroup	0.549	1	0.459
Condition	0.678	1	0.410
ProblemType	9.851	2	0.007
SubGroup:Condition	0.276	1	0.599

Figure 3 in Manuscript

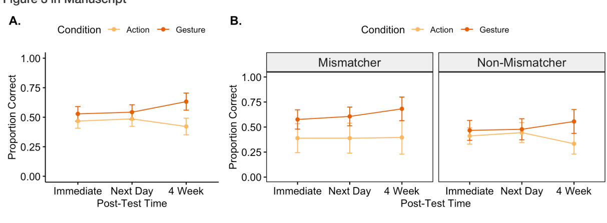

Study 2: AG vs GA Training

Post-test performance over time for action->gesture (AG, dark purple) and gesture->action (GA, light purple) instruction.

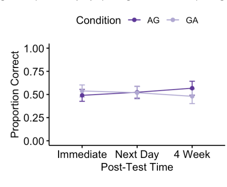

Condition	PostTestTime	meanProportionCorrect
AG	Immediate	0.491
AG	Next Day	0.524
AG	4 Week	0.568
GA	Immediate	0.538
GA	Next Day	0.519
GA	4 Week	0.480

Study 2 - Primary Analyses

Logistic Regression

```
S2_Primary <-
  glmer(Correct ~ Condition*PostTestTime_Scaled*ProblemType + (1|SubID),
        data=S2,
        family=binomial, glmerControl(optimizer="bobyqa", optCtrl=list(maxfun=100000)))
```

	Chisq	Df	Pr(>Chisq)
Condition	0.085	1	0.771
PostTestTime_Scaled	1.081	1	0.298
ProblemType	92.350	2	0.000
Condition:PostTestTime_Scaled	12.377	1	0.000
Condition:ProblemType	2.092	2	0.351
PostTestTime_Scaled:ProblemType	24.814	2	0.000
Condition:PostTestTime_Scaled:ProblemType	0.751	2	0.687

Follow-Up Logistic Regressions

AG Training over Time:

```
AG_TimeProbType <-
  glmer(Correct ~ PostTestTime_Scaled*ProblemType +  (1|SubID),
        data=subset(S2, Condition=="AG"),
        family=binomial, glmerControl(optimizer="bobyqa", optCtrl=list(maxfun=100000)))
```

	Chisq	Df	Pr(>Chisq)
PostTestTime_Scaled	9.048	1	0.003
ProblemType	57.228	2	0.000
PostTestTime_Scaled:ProblemType	12.286	2	0.002

	Estimate	Std. Error	z value	Pr(>|z|)
(Intercept)	2.086	0.717	2.910	0.004
PostTestTime_Scaled	-0.244	0.281	-0.867	0.386
ProblemTypeFormB	-2.800	0.370	-7.558	0.000
ProblemTypeFormC	-2.461	0.357	-6.885	0.000
PostTestTime_Scaled:ProblemTypeFormB	1.400	0.404	3.468	0.001
PostTestTime_Scaled:ProblemTypeFormC	0.905	0.392	2.310	0.021

GA Training over Time:

```
GA_TimeProbType <-
  glmer(Correct ~ PostTestTime_Scaled*ProblemType + (1|SubID),
        data=subset(S2, Condition=="GA"),
        family=binomial, glmerControl(optimizer="bobyqa", optCtrl=list(maxfun=100000)))
```

	Chisq	Df	Pr(>Chisq)
PostTestTime_Scaled	1.887	1	0.170
ProblemType	37.591	2	0.000
PostTestTime_Scaled:ProblemType	13.284	2	0.001

	Estimate	Std. Error	z value	Pr(>|z|)
(Intercept)	1.326	0.624	2.127	0.033
PostTestTime_Scaled	-1.107	0.290	-3.820	0.000
ProblemTypeFormB	-2.197	0.338	-6.492	0.000
ProblemTypeFormC	-1.744	0.328	-5.316	0.000
PostTestTime_Scaled:ProblemTypeFormB	1.275	0.386	3.304	0.001
PostTestTime_Scaled:ProblemTypeFormC	1.216	0.385	3.154	0.002

Immediate Post-Test, AG vs GA:

```
S2_T1 <- glmer(Correct ~ Condition + ProblemType +  (1|SubID),
               data=subset(S2, PostTestTime=="Immediate"),
               family=binomial, glmerControl(optimizer="bobyqa", optCtrl=list(maxfun=100000)))
```

	Chisq	Df	Pr(>Chisq)
Condition	0.208	1	0.648
ProblemType	48.927	2	0.000

24-hr Delayed Post-Test, AG vs GA:

```
S2_T2 <- glmer(Correct ~ Condition + ProblemType +  (1|SubID),
               data=subset(S2, PostTestTime=="Next Day"),
               family=binomial, glmerControl(optimizer="bobyqa", optCtrl=list(maxfun=100000)))
```

	Chisq	Df	Pr(>Chisq)
Condition	0.000	1	0.991
ProblemType	40.723	2	0.000

4-Week Delayed Post-Test, AG vs GA:

```
S2_T3 <- glmer(Correct ~ Condition + ProblemType +  (1|SubID),
               data=subset(S2, PostTestTime=="4 Week"),
               family=binomial, glmerControl(optimizer="bobyqa", optCtrl=list(maxfun=100000)))
```

	Chisq	Df	Pr(>Chisq)
Condition	1.342	1	0.247
ProblemType	11.706	2	0.003

Study 2 - Secondary Sub-Group Analysis

Study 2 Sub-Group Post-Test Performance over Time:

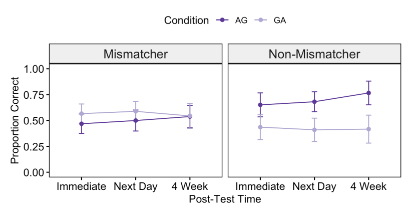

Study 2 Sub-Group Breakdown:

```
##
## ----------- ---------- ------------ ---------------- ------------- ----------- -------
##                SubGroup   Mismatcher   Non-Mismatcher   No Gesturer   Uncodable   Total
##   Condition
##          AG                       16               11             7           2      36
##          GA                       15               13             7           0      35
##       Total                       31               24            14           2      71
## ----------- ---------- ------------ ---------------- ------------- ----------- -------
```

Logistic Regression:

```
S2_SubGrpReg <- glmer(Correct ~ SubGroup*Condition*PostTestTime_Scaled +
                        PostTestTime_Scaled:ProblemType +  (1|SubID),
                      data=S2sub, family=binomial,
                      glmerControl(optimizer="bobyqa", optCtrl=list(maxfun=100000)))
```

	Chisq	Df	Pr(>Chisq)
SubGroup	0.070	1	0.792
Condition	1.331	1	0.249
PostTestTime_Scaled	3.025	1	0.082
SubGroup:Condition	4.279	1	0.039
SubGroup:PostTestTime_Scaled	0.571	1	0.450
Condition:PostTestTime_Scaled	4.584	1	0.032
PostTestTime_Scaled:ProblemType	21.662	2	0.000
SubGroup:Condition:PostTestTime_Scaled	0.008	1	0.929

Follow-Up Logistic Regressions:

Non-Mismatchers - AG vs GA over Time:

```
AGGA_NonMMGrp <- glmer(Correct ~ Condition*PostTestTime_Scaled + PostTestTime_Scaled:ProblemType + (1|SubID),
                       data=subset(S2sub, SubGroup=="Non-Mismatcher"),
                       family=binomial, glmerControl(optimizer="bobyqa", optCtrl=list(maxfun=100000)))
```

	Chisq	Df	Pr(>Chisq)
Condition	3.950	1	0.047
PostTestTime_Scaled	2.954	1	0.086
Condition:PostTestTime_Scaled	1.776	1	0.183
PostTestTime_Scaled:ProblemType	2.535	2	0.282

Mismatchers - AG vs GA over Time:

```
AGGA_MMGrp <- glmer(Correct ~ Condition*PostTestTime_Scaled + PostTestTime_Scaled:ProblemType + (1|SubID),
                    data=subset(S2sub, SubGroup=="Mismatcher"),
                    family=binomial, glmerControl(optimizer="bobyqa", optCtrl=list(maxfun=100000)))
```

	Chisq	Df	Pr(>Chisq)
Condition	0.236	1	0.627
PostTestTime_Scaled	0.752	1	0.386
Condition:PostTestTime_Scaled	2.556	1	0.110
PostTestTime_Scaled:ProblemType	21.214	2	0.000

Figure 4 in Manuscript

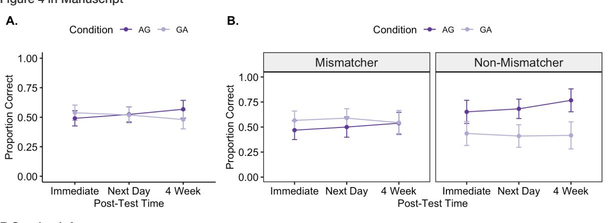

R Session Info

```
sessionInfo()
```

```
## R version 3.6.1 (2019-07-05)
## Platform: x86_64-apple-darwin15.6.0 (64-bit)
## Running under: macOS Mojave 10.14.6
##
## Matrix products: default
## BLAS:   /Library/Frameworks/R.framework/Versions/3.6/Resources/lib/libRblas.0.dylib
## LAPACK: /Library/Frameworks/R.framework/Versions/3.6/Resources/lib/libRlapack.dylib
##
## locale:
## [1] en_US.UTF-8/en_US.UTF-8/en_US.UTF-8/C/en_US.UTF-8/en_US.UTF-8
##
## attached base packages:
## [1] stats     graphics  grDevices utils     datasets  methods   base
##
## other attached packages:
##  [1] summarytools_0.9.8 cowplot_1.0.0      knitr_1.28         broom_0.5.6
##  [5] plyr_1.8.6         car_3.0-7          carData_3.0-3      dplyr_1.0.2
##  [9] lme4_1.1-21        Matrix_1.2-17      ggpubr_0.4.0       ggplot2_3.3.3
## [13] tidyr_1.1.2
##
## loaded via a namespace (and not attached):
##  [1] Rcpp_1.0.5         lubridate_1.7.9.2  lattice_0.20-38    digest_0.6.27
##  [5] R6_2.5.0           cellranger_1.1.0   backports_1.2.1    evaluate_0.14
##  [9] highr_0.8          pillar_1.4.7       rlang_0.4.9        curl_4.3
## [13] readxl_1.3.1       minqa_1.2.4        data.table_1.12.6  nloptr_1.2.1
## [17] magick_2.5.2       checkmate_2.0.0    rmarkdown_2.1      labeling_0.3
## [21] splines_3.6.1      stringr_1.4.0      foreign_0.8-71     pander_0.6.3
## [25] munsell_0.5.0      compiler_3.6.1     xfun_0.13          pkgconfig_2.0.3
## [29] base64enc_0.1-3    tcltk_3.6.1        htmltools_0.5.0    tidyselect_1.1.0
## [33] tibble_3.0.4       rio_0.5.16         codetools_0.2-16   matrixStats_0.57.0
## [37] crayon_1.3.4       withr_2.3.0        MASS_7.3-51.4      grid_3.6.1
## [41] nlme_3.1-140       gtable_0.3.0       lifecycle_0.2.0    magrittr_2.0.1
## [45] scales_1.1.0       zip_2.1.1          stringi_1.5.3      farver_2.0.1
## [49] ggsignif_0.6.0     pryr_0.1.4         ellipsis_0.3.1     rapportools_1.0
## [53] generics_0.1.0     vctrs_0.3.6        boot_1.3-22        openxlsx_4.1.4
## [57] tools_3.6.1        forcats_0.4.0      glue_1.4.2         purrr_0.3.4
## [61] hms_0.5.2          abind_1.4-5        yaml_2.2.1         colorspace_1.4-1
## [65] rstatix_0.6.0      haven_2.2.0
```
